# A Modern Miracle

**Published:** 1890-07-05

**Authors:** 


### The Hospital

A Weekly Journal of

Science, Medicine, Nursing, and Philanthropy.

*For Hospitals, Asylums, Medical Practitioners, Students, Nurses, Families, Parishes, Congregations, and the Charitable Public.*

Vol. VIII.—No. 197.] [July 5, 1890.

### A Modern Miracle.

A correspondent sends to us the following story: "Yesterday I was conversing with a Franciscan Friar on the subject of miracles. In proof of his theory that cures were effected by 'Our Lady of Lourdes,' he told me an incident in his own life. When a little boy, said he, I had an attack of croup. I was carried out of the church black in the face. A doctor was sent for, who, as soon as he saw me, ran for his tools to put a silver pipe in my throat. While he was gone my mother poured into my mouth a spoonful of water consecrated to Our Blessed Lady. Immediately I spat up a large piece of black skin; I recovered, and when the doctor came back he said, 'Why, what have you been doing?' There was then, I suppose, a family scene of rejoicing, as the doctor was a devout man, and the patient, eight years old, was there and then devoted to the priesthood. His father made a vow that he would attend his celebration of High Mass, which vow he fulfilled three years ago. Now, says our correspondent, I saw two tracheotomy cases in the London Hospital, but I never saw any black skin spat up, and I never heard that black skin was a feature in croup. But that the child recovered is manifest. My question is, was there anything supernatural or unusual in the recovery? I do not think the Friar is a man who would suffer himself to be imposed upon or would impose upon others."

All people who take a broad and intelligent interest in the history and destiny of man are aware of the importance attached to miracles by two opposite and antagonistic schools of thought. The Church, using that term in its widest sense, affirms the fact of miracles and sees no difficulty in them. Certain speculative thinkers, men of undoubted intellectual capacity, on the other hand, not only deny the fact of miracles but affirm their *à priori* impossibility. At bottom the real position of Hume was that miracles could not take place and that therefore they never had taken place. A more unphilosophic position than this no man, not even a philosopher, ever took up. That David Hume or any other person possessed of merely human faculties, is able to say what can and what can not take place in this practically infinite universe is a proposition which carries its own contradiction in its face. No man who has ever seen a sperm cell under the microscope and has thereafter made a minute dissection of the adult human body will speak much of impossibilities in this world of wonders. That an egg filled with a white fluid and a semi-solid yolk should in three weeks, under the mere influence of heat, develop into a living bird with muscular body and wings, with brain and spinal cord, with heart, lungs, and liver, with eyes, ears, and taste—if that is not a miracle, what is it? Until David Hume or at least his followers can explain that for us we shall not attach too much importance to their *à priori* antagonism to miracles.

But now, having affirmed what we consider to be the truly scientific attitude of the mind on the question, we will discuss with sober impartiality our correspondent's story. For we cannot but think that those who are too ready to see miracles and to strive to impose them upon their neighbours do more harm to reasonable religion than sceptics. The story is that of a little boy, eight years old, who had an attack of croup, and was carried out of church black in the face. But what was a boy suffering from croup doing in church at all? If he had really had croup he would have been in bed, and not at church. True, croup often comes on suddenly, in the middle of the night; but we never heard of a case of a child, in apparently his ordinary health, developing in a moment and without any preliminary symptom whatever, signs of the most urgent and immediate suffocation. The medical mind would, therefore, at once dismiss altogether the notion of croup as the cause of the child's alarming attack. But if it was not croup, what was it? Here the "black skin" which presents such difficulties to our correspondent, comes to our aid. The membrane of croup is not a black skin in any sense, but has an ash grey or dirty yellow colour. Of course, certain drugs darken this membrane exactly as they blacken the tongue; and if the boy had been under treatment at home for two or three days instead of being at church in his ordinary health, the croup membrane might have looked like a black skin. But even then the mere bringing up of the membranous substance after drinking a little consecrated water would not have proved that the water was the cause of the expectoration, for croupous membrane is often coughed up after drinking plain water or medicine, and still oftener it comes up, when it is detached and ready, by the ordinary efforts of nature, and without drinking anything at all.

The "black skin" remains to be accounted for. There are many possible explanations of its presence in the throat or windpipe. The boy might have been eating grapes, or black cherries, or French plums, or other like fruit, and some portion of unmasticated rind might have lodged between the jaw and the cheek, or in a depression of the tonsil at the time of eating, and slipped down into the windpipe an hour or two afterwards when the boy was in church; or he might have eaten any of the things mentioned, even so long as a few days before the occurrence in church, and a piece of black fruit rind might have passed into the windpipe, and rapidly fallen into a large bronchus without producing more than a little momentary irritation at the time. This becoming displaced might easily close up the largest bronchial tubes on one side, and thus give rise to sudden difficulty of breathing, with all the external appearances of impending suffocation. The violent expulsory efforts following upon the drinking of the consecrated water would, in all probability, have come on just the same if no water had been drunk. Cases such as we have here supposed are quite common in the history of medicine, and do not in any way demand the importation of a miracle for their explanation.

There is no need whatever to question the Franciscan Friar's good faith in the matter; but there is some reason to doubt whether the doctor was quite so well acquainted with his business as he ought to have been. What the clergy and all religious teachers should understand is, that science can never accept a religion which is not entirely rational. There are two leading ideas prevalent in clerical minds. One is that religion is to be imposed upon the people by ecclesiastical authority. The Church speaks—the people must hear, believe, and obey. The other looks upon religion as a subject of instruction and persuasion. If a man is to be made religious he must be dealt with exactly as he is dealt with when he is made scientific; he must be instructed, persuaded, encouraged, and helped by precept and example, and his religious character gradually formed by all the other methods of the teacher. To take dogmas to a scientific man and to insist that he shall accept them on the sole authority of a Church, whether that Church be Catholic, Protestant, or sectarian, and to try to buttress the dogmas by doubtful miracles is to deliberately drive the scientific man into an attitude of scepticism, and, sometimes, even of avowed hostility. Nothing is more injurious to the cause of true religion than this very claim of ecclesiastical authority. At the present time science is, so to speak, a part of the very air we breathe. The facts of science, and, to some extent, her methods, are taught even in our elementary schools. A spirit of inquiry and criticism is thereby awakened, and it is a ghost that will not be laid by authority. We do not object to dogma as dogma; for, in our judgment, Mr. Mathew Arnold notwithstanding, dogma is as essential to clearness of teaching as an architect's plan is to the squareness and perpendicularity of a house. Dogma as a framework, and as a starting-point, is necessary both in religion and in science; but dogma to be received on authority alone, and never to be questioned, is the death of intellectual life and of reasonable religion. It is not to be tolerated for an hour by any believer in Newton or St. Paul. Possibly it may be considered that we have wandered a little from our text, but we elected to make our correspondent's letter the subject of an article for that very purpose. Reasonable religion we hold to be the soul of philosophy, and the highest life of both the intellectual and the common man. Any line of thought or way of life that seems to decline therefrom we consider it a part of our duty both to point out and to resolutely oppose.

